# Experimental Characterisation of Differently Composed Thrombus Entities with Spectral-Detector-CT

**DOI:** 10.3390/neurolint18020038

**Published:** 2026-02-21

**Authors:** Schekeb Aludin, Agreen Horr, Lars-Patrick Schmill, Carmen Wolf, Olav Jansen, Bodo Kurz, Julian Andersson, Svea Seehafer, Naomi Larsen, Patrick Langguth, Jens Trentmann

**Affiliations:** 1 Department of Radiology and Neuroradiology, University Hospital of Schleswig-Holstein, 24105 Kiel, Germany; agreen.horr@uksh.de (A.H.); lars-patrick.schmill@uksh.de (L.-P.S.); svea.seehafer@uksh.de (S.S.);; 2 Institute of Anatomy, Christian-Albrechts-University Kiel, 24118 Kiel, Germany

**Keywords:** acute ischemic stroke, spectral-detector-CT, dual-layer-CT, spectral-CT, thrombus, phantom study

## Abstract

**Background/Objectives**: Thrombus composition influences the success of endovascular therapy in stroke, but conventional CT is limited in determining it. Spectral-detector-CT (SDCT) can apply material-decomposition and virtual monoenergetic (MonoE) imaging, which may provide a way to gain information on thrombus composition. This experimental study aimed to evaluate the differentiability of heterogeneous thrombi with variable red blood cell (RBC) content using SDCT. **Methods**: Ten thrombus entities with different compositions on RBC and plasma, thus fibrin content, were manufactured (volumetric RBC%/Plasma% = 90/10; 80/20; 70/30; 60/40; 50/50; 40/60; 30/70; 20/80; 10/90; 5/95) and scanned in an SDCT. Conventional Hounsfield-unit (HU) values, spectral electron density (ED), effective atomic number (Z-effective) and HU in MonoE maps ranging from 40– to 200 keV were evaluated for thrombus differentiation. **Results**: Conventional HU increased with RBC content, allowing us to differentiate the entities (*p* < 0.001). ED values also increased with RBC content and allowed for differentiation too (*p* < 0.001). Z-effective values showed no differences among the different entities (*p* > 0.05). Regarding the mass-attenuation curves from 40 to 200 keV the different thrombi showed a similar curve progression with highest HU values at 40 and lowest at 200 keV. The thrombi could be distinguished overall at each monoenergetic level by HU (*p* < 0.001 for each level). The absolute decrease in HU between 40 and 200 keV was thereby not significantly different between the different entities, but the relative decrease was, as it was more pronounced in thrombi with lower RBC content (*p* < 0.001). **Conclusions**: Spectral CT enables differentiation between thrombi with different RBC and fibrin contents by means of ED or analysis of the mass-attenuation curve. This offers alternative possibilities that go beyond characterisation based on CT-density alone. The additional inclusion of spectral parameters in thrombus diagnostics could therefore improve diagnosis and treatment.

## 1. Introduction

Thrombus is the most prevalent cause of vascular occlusion in acute ischemic stroke (AIS), and it is well known that thrombi in general exhibit a structural heterogeneity that depends on the vascular circumstances [1,2,3]. For example, venous or cardiac thrombi originating from the left atrial appendage tend to form under static conditions and are rich in red blood cells (RBCs). Arterial thrombi, on the other hand, are more likely to form under high shear forces and have a lower RBC but higher fibrin content [2,3,4]. These components hereby have a decisive influence on the thrombus’ mechanical properties as fibrin-rich thrombi are more rigid, while RBC-rich thrombi are soft and vulnerable to mechanical manipulation [5,6]. Previous clinical studies have demonstrated that this structural and histological heterogeneity is also prevalent in thrombi extracted from patients with AIS [7,8,9]. Interestingly, the thrombi’s composition and associated mechanical properties appear to have a relevant influence on the therapeutic efficacy of mechanical recanalisation performed with different endovascular thrombectomy techniques [10,11,12,13,14,15]. RBC-rich thrombi hereby tend to be softer and can be removed well using aspiration thrombectomy. In contrast, fibrin-rich thrombi tend to be more rigid and can be difficult to remove using aspiration thrombectomy, which is why multiple manoeuvres may be necessary. In such cases, stent retriever thrombectomy or combined approaches appear to be more suitable as they can better mechanically grab and retract the thrombus [10,11,12,13,14,15]. Regarding other therapeutic methods such as intravenous thrombolysis, it also appears that RBC-rich thrombi are more vulnerable to the thrombolytic agent and can be better dissolved by it. In contrast, fibrin-rich thrombi are more resistant, and thrombolytic therapy appears to be associated with poorer results in these cases [14,16,17]. Regarding these circumstances, pre-interventional knowledge of the thrombus composition and a corresponding estimation of its mechanical properties would be advantageous for treatment-planning but is not yet available from routine clinical diagnostics [9,18].

CT is the most widely used imaging modality for diagnosing AIS, and conventional scanners are thereby typically referred to as single-energy CT (SECT) [19]. They measure X-ray attenuation at one energy level and express it as HU values [20]. In stroke imaging, thrombi can be visualised by CT. E.g., it is sometimes possible to detect a thrombus in non-contrast enhanced CT as it offers a higher radiopacity than blood, which is then referred to as a “hyperdense artery sign” [21,22,23]. Several studies suggest that this phenomenon correlates with an increased RBC content, as RBCs contain iron-rich haemoglobin. In this way, this imaging phenomenon allows a rough estimation of the thrombus composition regarding its RBC content [24,25]. However, SECT is restricted to the detection of only one X-ray energy, which leads to significant limitations in the differentiation of materials that exhibit similar attenuation at this singular energy level, e.g., iron and calcium [20]. In recent years, CT technology has evolved, leading to new techniques such as spectral CT (SCT), which overcome this limitation. SCT scanners can detect different X-ray energies and can be applied by using various techniques for image acquisition. A distinction is hereby made between source-based and detector-based techniques [20,26,27]. With source-based techniques, a decision on whether spectral data should be acquired must be made before the scan. These include dual-source CT, which uses two source-detector systems, and kV-switching CT, which alternates the energy of the X-rays at a high speed [20,26,27]. With detector-based techniques in contrast, there is no need to decide in advance whether spectral data should be acquired, as this is done automatically with every scan. These include spectral-detector-CT (SDCT), which uses a layered detector system that can detect two X-ray energies simultaneously, and photon-counting CT, which uses semi-conductive detector materials that enable a direct measurement of photon energy [20,26,27]. By using such techniques, a more extensive spectrum of energy levels is covered, which enables a valid, distinct characterisation of materials, referred to as material decomposition, as well as the derivation of spectral parameters such as electron density (ED) or the effective atomic number (Z-effective) [20,27,28,29]. Given the clinical relevance of thrombus composition and the limitations of SECT, SCT appears to be an interesting application that could potentially make a valuable contribution to pre-interventional thrombus characterisation.

In this experimental study, the spectral behaviour of artificially manufactured, standardised thrombi with variable RBC and fibrin content were investigated using an SDCT. This study’s aim was to evaluate whether spectral parameters such as ED, Z-effective or analysis of the mass-attenuation curves can be used to characterise the entities compared to conventional CT density.

## 2. Materials and Methods

### 2.1. Thrombus Preparation and Imaging Phantom

**Figure 1:**
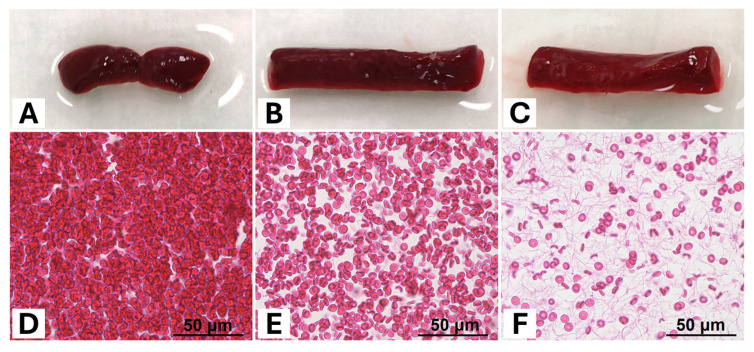
(**A**–**C**) Exemplary macroscopic images of a selection of different thrombi according to their red blood cell (RBC) content (RBC content (**A**) = 90%, (**B**) = 50%, (**C**) = 5%). Although their RBC content is quite different, the thrombi all appear red with only a slight lightening of their colour with decreasing RBC content. (**D**–**F**) Representation of histological tissue sections of the corresponding thrombi as in (**A**–**C**) stained with haematoxylin–eosin (slice thickness 4–5 μm, 40× magnification) (RBC content (**D**) = 90%, (**E**) = 50%, (**F**) = 5%). While there is a very high density of RBCs at 90% in (**D**), this decreases in (**E**,**F**). With decreasing RBC content, the network of thin fibrin strands can be increasingly better delineated, as the relative proportion of plasma in the test preparation is likewise increased.

Ten types of thrombi with different compositions were prepared from human blood for imaging. The technique and applicability of artificially generated thrombi have been validated in previous studies [30,31,32]. Blood was collected from male volunteers who gave written consent to participate. Coagulopathies or the use of anticoagulant medication in the previous 10 days were excluded and queried beforehand. Venous blood was drawn, mixed with a 9:1 anticoagulant citrate solution and centrifuged at 1800× *g* for 10 min to separate plasma (PL) and corpuscular components, representing mainly RBC. Ten different mixtures were prepared, which constituted a series of experiments. Each had a volume of 1 mL and consisted of a specific ratio of RBC and PL (RBC%/PL%): (1) 90/10; (2) 80/20; (3) 70/30; (4) 60/40; (5) 50/50; (6) 40/60; (7) 30/70; (8) 20/80; (9) 10/90; (10) 5/95. The preparations were placed in small polyethylene tubes of 5 mm diameter. Calcium-chloride solution was added to restore coagulability. The plastic tubes were sealed at both ends and incubated at room temperature for two hours to allow thrombi to form (Figure 1). During this, the tubes were rotated to prevent corpuscular sedimentation, so that thrombi with a homogeneous structure were created. Ten thrombi were produced from each of the ten mixtures, giving a total number of 100 thrombi.

**Figure 2:**
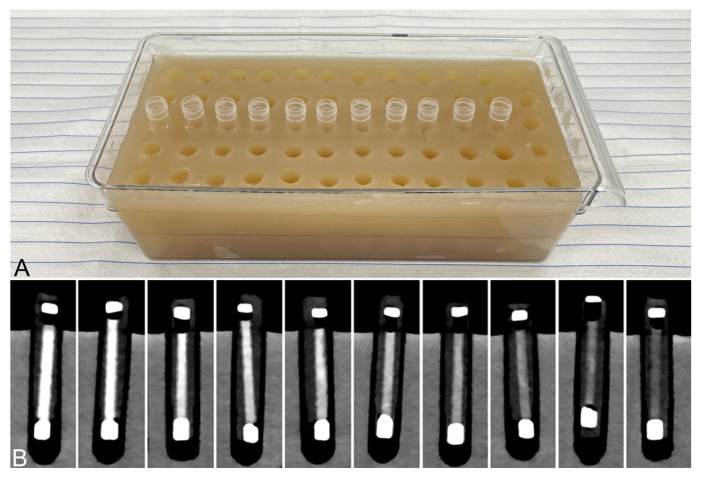
(**A**) Agarose-gel imaging phantom with slots for inserting sample vials. An exemplary series of vials is inserted. (**B**) Conventional computed tomographic images of the different thrombus entities within the imaging phantom. The thrombi are inside polyethylene tubes, which are sealed at both ends. These in turn are placed in tubes filled with an isotonic saline solution and surrounded by the agarose-gel matrix. The thrombi are arranged according to their RBC content (from left to right: 90%, 80%, 70%, 60%, 50%, 40%, 30%, 20%, 10%, 5%).

The thrombi within the sealed tubes were placed in bigger tubes filled with isotonic sodium-chloride solution. These were in turn placed into an agarose-gel imaging phantom, which had a conventional CT density of approximately 35–45 HU, corresponding to human soft tissue (Figure 2A). To each series of tested thrombi, an additional tube of uncoagulated blood was added for comparison.

### 2.2. Image Acquisition

Imaging was performed on an SDCT (IQon, PHILIPS Healthcare, Best, The Netherlands) with a scan protocol for a non-contrast enhanced scan of the cranium. The acquisition parameters were 225 mAs tube current, 120 kVp tube voltage, 64 × 0.625 mm collimation, 0.39 pitch and 0.31 increment. Conventional images were reconstructed at a slice thickness of 0.8 mm (Figure 2B). In addition, spectral-base-image (SBI) datasets with the same slice thickness were generated from which spectral maps could be derived using the manufacturer’s software (IntelliSpace Portal^®^ 11, PHILIPS Healthcare, Best, The Netherlands). The following spectral maps were generated: (1) ED; (2) Z-effective; (3) virtual monoenergetic (MonoE) maps ranging from 40 to 200 keV (in steps of 20 keV).

### 2.3. Image Analysis

**Figure 3:**
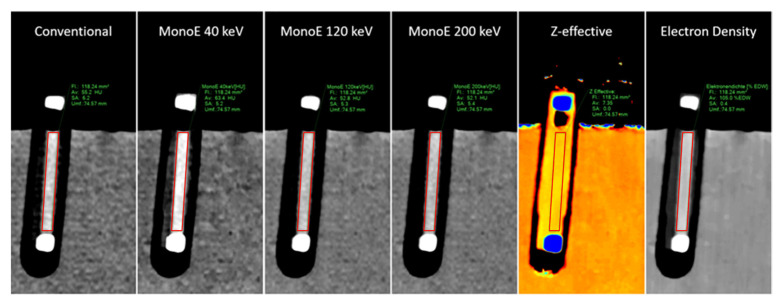
Depiction of a thrombus with 50% RBC content in the various image maps (as indicated, from left to right: conventional, virtual monoenergetic images at 40 keV (MonoE 40 keV), 120 keV (MonoE 120 keV) and 200 keV (MonoE 200 keV), effective atomic number (Z-effective) and electron density). In addition, exemplary ROI marking (as red line) in each of the thrombus in all image maps as part of the image analysis.

To obtain quantitative parameters, two independent readers (two radiologists with 5 and 6 years of experience in CT imaging), who were blinded to the composition of the thrombi, placed a region of interest (ROI) in each thrombus. This ROI was placed in the mid-plane of each thrombus in its longitudinal alignment (i.e., a lengthwise section through the cylindric thrombus) along its boundaries. The ROI was automatically transferred to all imaging maps, ensuring their comparability (Figure 3).

The CT density of each thrombus was quantified in HU in the conventional images and in the MonoE maps. The ED and Z-effective values were measured in the respective spectral maps. The thrombi were compared to each other and to the uncoagulated blood sample with regard to their quantitative values measured in the conventional images, the ED maps and Z-effective maps. Regarding the MonoE maps, the differentiability was evaluated at each MonoE level. Furthermore, the curve progression of the mass-attenuation curves across the different MonoE levels was evaluated for the different thrombi. Thereby, in each thrombus entity the difference in HU values between the 40 keV and the 200 keV images was calculated. The difference was given in absolute HU values and as a percentage ratio. Based on this, it was evaluated whether the thrombus entities could be differentiated by absolute or relative differences at these different MonoE levels. Besides these analyses on the differentiability of the individual entities, an explicit comparison between RBC-rich and RBC-poor thrombi was also carried out. For this purpose, the thrombi were summarised into two groups according to their RBC content. The thrombi with an RBC content between 50 and 90% were categorised as the “RBC-rich” group, and the thrombi with 5–40% as the “RBC-poor” group. Receiver operating characteristic (ROC) and area under the curve (AUC) analysis was performed for these two groups’ differentiability in conventional images, ED, Z-effective and each MonoE level. It was evaluated in which map the differentiation between these two groups was best.

### 2.4. Histological Evaluation

Representative thrombi from each entity were fixed in a 4% paraformaldehyde solution (final concentration) for a period of 48 h. Subsequently, the thrombi were prepared for histological evaluation by extracting water and embedding them in paraffin wax. Each specimen was cut into sections of 4–5 μm thickness. These were stained with haematoxylin–eosin, and high-resolution images were obtained using a slide scanner (Aperio CS2, Leica, Germany). The histological sections were visually examined for the microscopic structure and the distribution and proportion of RBC and fibrin.

### 2.5. Statistics

Statistical analysis was performed using the jamovi project for Windows (version 2.2.5, Sydney, Australia, 2021). Visualisation of data was performed using Microsoft Excel (version 2604). The HU values of the thrombus entities in the conventional images as well as the ED values and the Z-effective values were summarised as mean values with standard deviation and visualised as box plots. The average HU values of the thrombus entities across different energy levels were presented. The absolute and percentage-wise HU decay from 40 keV to 200 keV were presented as box plots. For all metric data, normal distribution was tested by using the Shapiro–Wilk test. Homogeneity of variances was tested using Levene’s test. The Kruskal–Wallis test and Dwass–Steel–Critchlow–Fligner test were employed to conduct statistical comparisons between the thrombus entities and uncoagulated blood. *p*-values are given in tables. Level of significance was set at 0.05 for all tests.

## 3. Results

### 3.1. Comparison of the Individual Thrombus Entities

#### 3.1.1. Conventional Image Analysis

**Figure 4:**
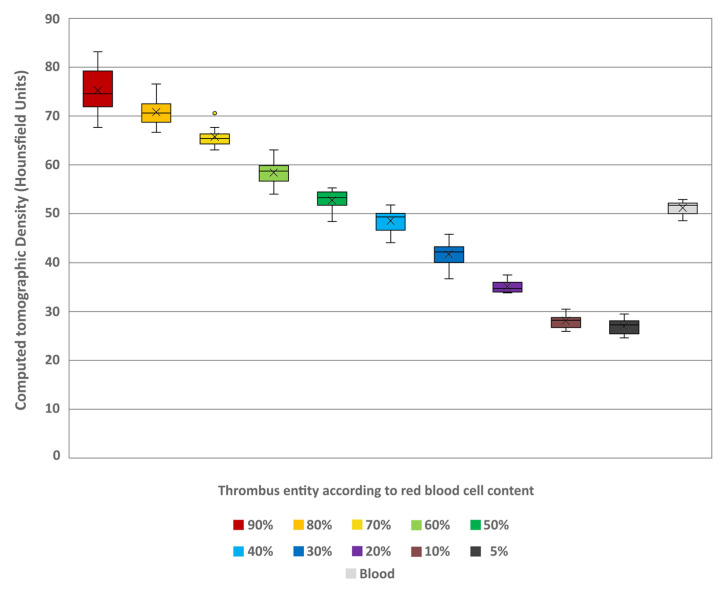
Boxplot diagram indicating the Hounsfield unit values of the different thrombus entities according to their red blood cell content and of a sample of uncoagulated blood in conventional images. The results of statistical testing are not given here; in this regard, reference is made to Table 1. Boxes show the median as horizontal line with the range from the 25th to 75th percentile. Whiskers show the range from minimum to maximum values. Mean values are indicated as cross. Outliers are indicated as dots.

The conventional density and spectral parameters could be measured in all samples. The interrater reliability was hereby excellent for all values (ICC = 0.955). The thrombus entities showed a notable decrease in conventional HU values (given in mean ± SD) with decreasing RBC content. The lowest values were at 5% (26.94 ± 1.7) and highest at 90% RBC content (75.24 ± 5.39) (Figure 4). The differences between the thrombus entities were found to be highly significant (H = 105.83; *p* < 0.001). A pairwise comparison revealed that the individual thrombus entities exhibited significant differentiability, with greater differences in RBC content resulting in improved differentiability (Table 1). Furthermore, comparison of the individual thrombus entities with uncoagulated blood (51.22 ± 1.49) also revealed significant differences in HU (Table 1). Notably, only the thrombi with 40% (48.57 ± 2.64) and 50% RBC content (52.78 ± 2.25) were not significantly different from the uncoagulated blood (*p* = 0.404 and *p* = 0.815, respectively).

#### 3.1.2. Spectral Image Analysis

**Figure 5:**
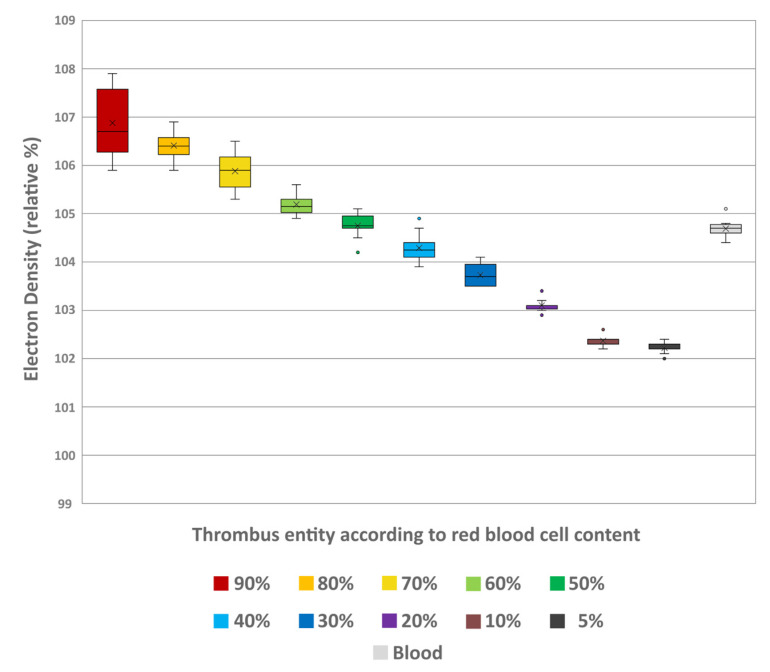
Boxplot diagram of the electron density values of the different thrombus entities according to their RBC content and uncoagulated blood. The results of statistical testing are not given here; in this regard, reference is made to Table 2. Boxes show the median as horizontal line with the range from the 25th to 75th percentile. Whiskers show the range from minimum to maximum values. Mean values are indicated as cross. Outliers are indicated as dots.

Spectral analysis of the thrombi showed a decrease in ED values with decreasing RBC content. The lowest values were at 5% (102.22 ± 0.11) and highest at 90% RBC content (106.88 ± 0.73) (Figure 5). The overall differences between the thrombus entities were highly significant (H = 105.10; *p* < 0.001). Pairwise comparison showed significant differentiability of individual thrombus entities, which were more pronounced with greater differences in RBC content (Table 2). Like in conventional imaging, comparison of the individual thrombus entities with the uncoagulated blood (104.69 ± 0.19) also revealed significant differentiability. Again, only the thrombi with 40% (104.29 ± 0.32) and 50% RBC content (104.75 ± 0.26) could not be distinguished from the uncoagulated blood sample (*p* = 0.236 and *p* = 0.998, respectively) (Table 2).

**Figure 6:**
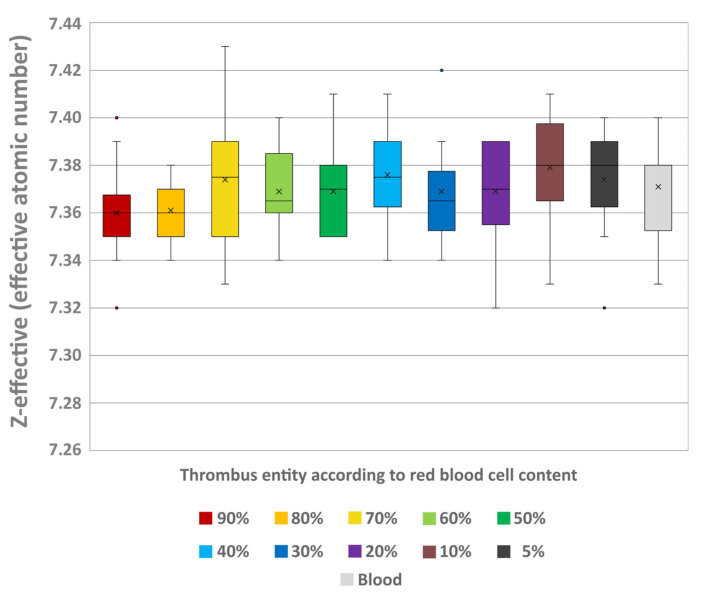
Boxplot of the effective atomic number (Z-effective) values of the different thrombus entities according to their RBC content and uncoagulated blood. Boxes show the median as horizontal line with the range from the 25th to 75th percentile. Whiskers show the range from minimum to maximum values. Mean values are indicated as cross. Outliers are indicated as dots.

Z-effective values showed no significant differences between the thrombi (H = 7.32; *p* = 0.695) and no trend of an increase or decrease was recognisable with differing RBC contents (e.g., 5% (7.37 ± 0.03) and 90% RBC content (7.36 ± 0.02)) (Figure 6). The individual testing also showed no significant differences between the thrombi and the uncoagulated blood sample (7.37 ± 0.02).

**Figure 7:**
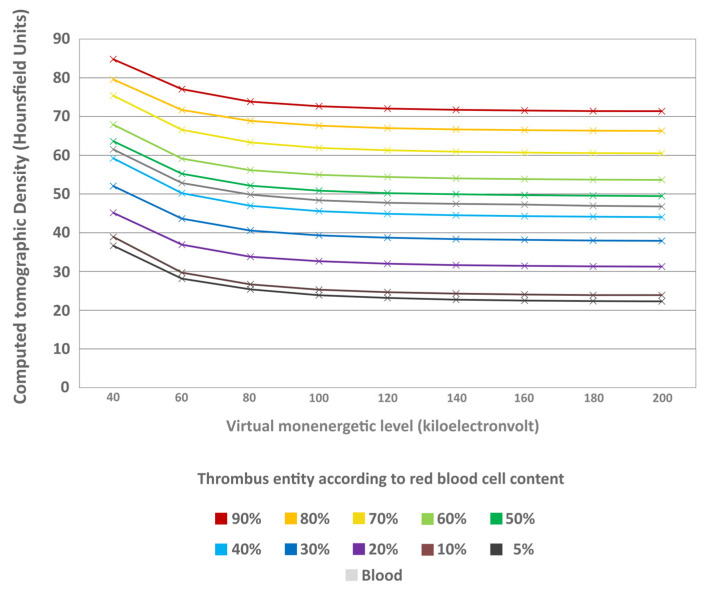
Mass-attenuation curves of the different thrombus entities. The CT density in Hounsfield units (y-axis) is given in relation to the keV spectrum of the virtual monoenergetic images between 40 keV and 200 keV in steps of 20 keV (x-axis). The thrombus entities are sorted according to their RBC content and highlighted in different colours (see image caption). Mean Hounsfield unit values at the different monoenergetic levels are indicated as cross for each thrombus entity. In addition, the values are also given for uncoagulated blood.

**Figure 8:**
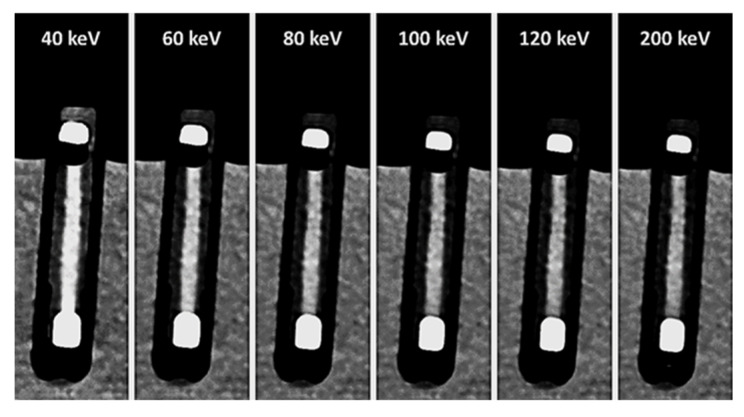
Depiction of the same thrombus (40% RBC content) in virtual monoenergetic images at six different energy levels: 40 keV, 60 keV, 80 keV, 100 keV, 120 keV and 200 keV. Particularly between 40 keV and 80 keV, a visual decrease in CT density is apparent. With higher energy levels, a further visual decrease in CT-density applies only to a very slight extent at best.

**Figure 9:**
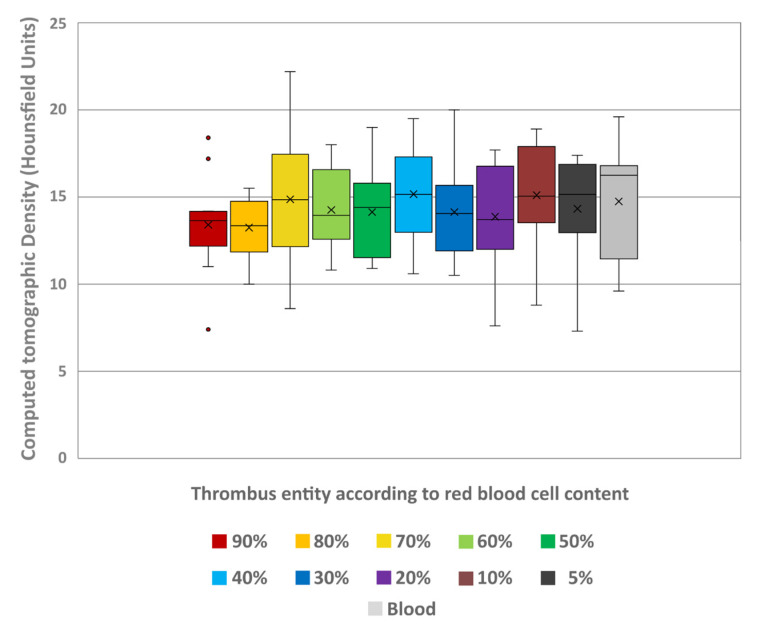
The boxplot shows the absolute difference in the thrombi’s CT density in Hounsfield unit (HU) values (y-axis) between the measured values in a virtual monoenergetic image at 40 keV and 200 keV ((HU values at 40 keV) − (HU values at 200 keV)). In addition, the values are also given for uncoagulated blood. Boxes show the median as a horizontal line with the range from the 25th to 75th percentile. Whiskers show the range from minimum to maximum values. Mean values are indicated as cross. Outliers are indicated as dots.

**Figure 10:**
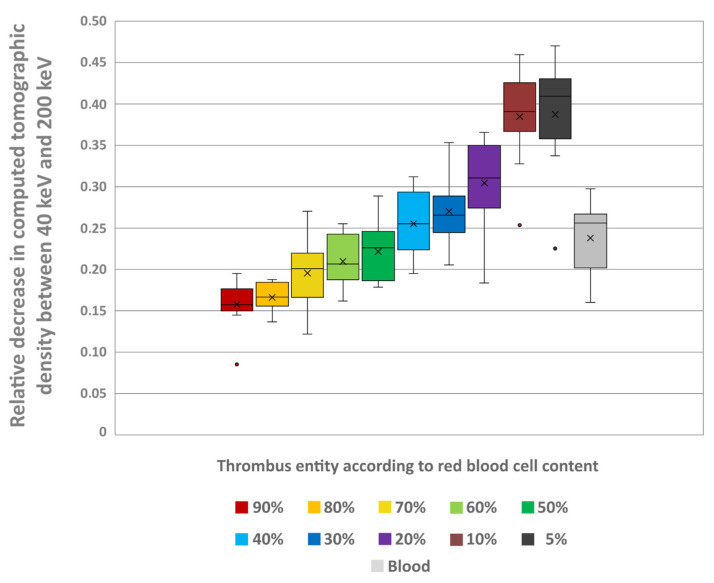
The boxplot shows the relative decrease in the thrombi’s CT density between the measured values in a virtual monoenergetic image at 40 keV and 200 keV (1 − (HU values at 200 keV)/(HU values at 40 keV)) for each thrombus entity. In addition, the values are also given for uncoagulated blood. The results of statistical testing are not given here; in this regard, reference is made to Table 3. Boxes show the median as horizontal line with the range from the 25th to 75th percentile. Whiskers show the range from minimum to maximum values. Mean values are indicated as cross. Outliers are indicated as dots.

The thrombi’s mass-attenuation curves between 40 keV and 200 keV offered highest HU values at 40 keV and lowest at 200 keV. Between these two maxima there was a decline in HU values, particularly between 40 keV and 80 keV (Figure 7). From 80 keV upwards, the decrease in HU values levelled off for all thrombus entities, which was also visually remarkable in the conventional CT images (Figure 8). The absolute decrease in HU values between 40 keV and 200 keV was not significantly different between the different thrombus entities (H = 4.66; *p* = 0.913) (Figure 9). However, there was a significant relative percentage decrease in HU values between 40 keV and 200 keV among the different entities (H = 77.94; *p* < 0.001) (Figure 10). The thrombi with a higher RBC content exhibited a lower overall percentage decrease in HU values (given in mean ± SD) between 40 keV and 200 keV (e.g., 90% = 15.77 ± 3.09) than those thrombi with a lower RBC content (e.g., 5% = 38.75 ± 6.99) (Figure 10). Paired comparison also revealed significant differences between the thrombus entities (Table 3). However, the significantly differentiable thrombi had greater differences in the RBC contents than the test results in the conventional or the ED images (Table 3).

### 3.2. Comparison Between “RBC-Rich” and “RBC-Poor” Thrombi

**Figure 11:**
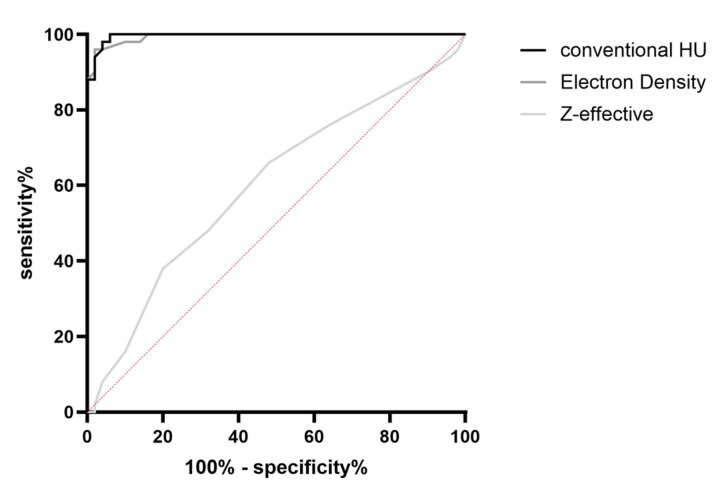
ROC diagram showing the differentiability between the groups of “RBC-rich” and “RBC-poor” thrombi by conventional Hounsfield units, electron density and effective atomic number (Z-effective).

Comparing the ROC curves between conventional HU, ED and Z-effective, the best differentiability between the “RBC-rich” and “RBC-poor” groups was demonstrated for conventional HU (AUC = 0.9962), followed by ED (AUC = 0.9942) and finally Z-effective, which demonstrated the lowest AUC value (AUC = 0.6012) (Figure 11). When comparing the different MonoE levels, the ROC curves and thus the AUC values were very similar for all levels (AUC at 40 keV = 0.944; 60 keV = 0.998; 80 keV = 0.9976; 100 keV = 0.9976; 120 keV = 0.9972; 140 keV = 0.9974; 160 keV = 0.9974; 180 keV = 0.9972; 200 keV = 0.9974).

### 3.3. Histology

The various thrombi had a similar histological basic structure, consisting of a network of fibrin strands with distributed RBCs therein (Figure 1). The thrombi did not demonstrate a higher ultrastructural order, and the RBCs appeared to be randomly arranged along the network. Regarding the different entities, the number of RBCs increased alongside the RBC content in the experimental mixture, which was visually evident as an increased appearance of RBCs and a rarefication of the fibrin strands. With decreasing RBC content in the sample preparation (and increasing proportion of PL), the strands became more pronounced, indicating an increasing proportion of fibrin compared to a decreasing proportion of RBC (Figure 1).

## 4. Discussion

Thrombus components such as RBC or fibrin content have a significant influence on its mechanics and the effectiveness of mechanical thrombectomy and thrombolytic therapy in AIS [5,6,7,8,9,10,11,12,13,14,16,17]. Pre-interventional knowledge of these by imaging would therefore be helpful in treatment planning but is limited with conventional CT [9,14,18]. However, new techniques such as SDCT with spectral imaging and analysis enable tissue characterisation beyond conventional density and could therefore offer new ways of thrombus characterisation [20,27]. In this study, ten thrombus entities with variable RBC content were created and scanned in an SDCT. The findings demonstrate that aside from conventional CT density alone, spectral CT enables differentiation of heterogeneous thrombi using spectral parameters.

The different thrombus entities could be distinguished by their conventional HU values, which almost steadily increased with their RBC content. This has also been described in previous studies, and the high CT density of iron in haemoglobin-containing RBCs is assumed to be the cause [6,31,32,33,34,35,36]. For example, Velasco et al. examined artificial thrombi and demonstrated a positive correlation of HU values and RBC content [36]. Similarly, Cahalane et al. also demonstrated a positive correlation between the RBC content of thrombi and their HU values in conventional images [6]. These findings are in line with the present study’s results and support them accordingly. In another study, Ding et al. also examined artificial thrombi and demonstrated significantly higher HU values in thrombi with 90% and 75% RBC content compared to 5%, 25% and 50%. Interestingly, however, this group described a plateau of the HU values in the range up to 50% RBC content, and a significant increase only occurred above this level [31]. In the current study, this specific circumstance was not observed, as the increase in HU values was present over the entire range between 5% and 90%. The reason for this discrepancy is unclear but may be due to methodological differences, e.g., the previous study used a different scanner type, which may be relevant in this regard [31]. Furthermore, differences in the imaging phantom or thrombus manufacturing could also be responsible. An important aspect could therefore be the natural degradation of thrombi over time. Previous studies have shown that a rapid change in haemoglobin or haematocrit can be detected in manufactured thrombi after a few hours to a few days [37,38]. This aspect was not systematically recorded in previous studies, and there is a lack of information on time intervals, which limits the comparability in this regard [6,31,32,36]. In the current study, we attempted to minimise this influence by keeping the time interval between thrombus preparation and scanning as short as possible (a maximum of up to 6 h). However, it must be noted that we did not include a dedicated measurement and documentation of the time interval in our study design. Accordingly, this should be considered a limitation here, and future studies should take this important aspect into consideration by systematically offering time intervals to improve methodological transparency and comparability. In view of the overall study situation, the present study’s findings regarding a positive relationship between the conventional CT density of thrombi and their RBC content are consistent with those of previous ones [6,32,35,36].

Besides conventional imaging, spectral imaging enabled the evaluation of the HU values for thrombus entities across the 40–200 keV spectrum, as well as derivation of material-specific mass-attenuation curves. Given that iron-binding haemoglobin in RBCs contains most of the iron in blood, it is reasonable to assume that its variable content may influence the mass-attenuation curves of thrombi [20,27,39,40]. The highest HU values were hereby observed at low MonoE levels for all thrombus entities, with a decrease towards higher levels. The differentiability of the thrombus entities was significant at all levels with very similar AUC values in the ROC analysis between RBC-rich and RBC-poor thrombi. There have been previous studies that investigated the HU-based differentiability of thrombi at different X-ray energies [31,32,41]. However, the study situation is ambiguous and offers variable results. Brinkjikji et al. evaluated artificial thrombi with different RBC contents in a dual-source CT and found an energy level of 80/140 kV to be the best for differentiation [32]. Ding et al. examined RBC-rich and RBC-poor thrombi in a photon-counting-detector CT and concluded that thrombi have the highest HU values and best differentiability at 50 keV, compared to conventional imaging [31]. In contrast, Panyaping et al. examined thrombi in a rapid kV-switching CT and concluded that the best differentiation was possible at 80 keV [41]. To a certain degree, these results are comparable with the present results, as they indicate that the highest HU values are found at low MonoE levels. However, better differentiation at a certain MonoE level is not evident, as the previous results appear to contradict each other to some extent. Additionally, our results also did not explicitly indicate improved differentiability at a certain MonoE level, as the AUC values differ only slightly. One reason for these discrepancies between the studies could be the use of heterogeneous scanners with different technical specifications [31,32,41]. Furthermore, the methodological aspects of the thrombi or the imaging phantom could also be responsible again, as each study has slightly different protocols in this regard. Accordingly, the comparability of the studies is limited, which should be considered when interpreting the results. However, in addition to evaluating a certain MonoE level, the present study’s dedicated analysis of the mass-attenuation curves indicated that the percentage-wise drop in HU values from 40 keV relative to 200 keV was significantly greater in thrombi with lower RBC content. To the best of our knowledge, this has not been described in previous in vitro studies and thus represents an innovative approach that specifically analyses the spectral CT behaviour of thrombi.

The thrombi were also evaluated using ED and Z-effective maps. ED describes the density of the negative charge in a volume and is expressed as a percentage of the negative charge density of water [28]. The present study showed that, as with conventional HU values, ED values of the thrombi increase with its RBC content and that it is possible to differentiate between the thrombi based on this. Prior studies have not investigated ED values for different thrombus entities in detail, so their use represents an innovative approach. In the broader field of thrombus imaging, Rodriguez-Granillo et al. evaluated the detectability of thrombi in general in ED images compared to conventional images and low MonoE images. However, their study did not include a dedicated analysis of the differentiability of different thrombus entities [42]. Recently, attempts have been made to analyse this parameter in uncoagulated blood samples [43]. Schulz et al. investigated the spectral CT properties of blood samples from anaemic patients in vitro, correlating the ED values of the samples with the haematocrit and haemoglobin values for anaemia diagnostics [43]. They were able to demonstrate that the ED of blood samples correlates positively with their haematocrit and haemoglobin content [43]. Uncoagulated blood samples naturally differ substantially from thrombi in terms of both histology and ultrastructure and are probably only comparable to a limited extent in this respect. However, there appear to be parallels, at least with artificially manufactured thrombi, in which the entire blood column coagulates and where there is no compositional change to the corpuscular components. This is also supported by the analysis of the Z-effective values, which describe the element-dependent effective nuclear charge [29,43]. Schulz et al. also compared the Z-effective values of their blood samples with the haematocrit value and the haemoglobin value, but no correlation was found [43]. Apart from this, Gassenhuber et al. recently analysed the Z-effective values of ex vivo thrombi and correlated them with the RBC content. They were unable to detect any significant differences between the thrombi [44]. A similar situation was found in the present study with the artificially manufactured thrombi, in which no correlation between the RBC content and Z-effective values could be demonstrated. Accordingly, the previously described correlations of ED and Z-effective regarding the content of haemoglobin and haematocrit (or RBC in ex vivo thrombi) appear to be transferable and consistent with the present study’s results with artificially manufactured thrombi. These findings contribute to the knowledge about the imaging characteristics of thrombi and could be used to estimate thrombus composition using imaging techniques.

This study has some limitations. The various thrombus entities were prepared using an artificial technique to generate thrombi with differing RBC content. This could also be demonstrated histologically, as the RBC content in the thrombi increased alongside that in the preparation mixture, while the fibrin network became more pronounced with increasing PL content in the preparation mixture. These results are consistent with those of previous studies in which artificial thrombi were used for imaging, and this distribution could be quantified histologically [30,31]. Although a dedicated histological quantification was not performed in the present study, the methodological approach and the histological characteristics still suggest a corresponding relationship. Another noteworthy observation was the random distribution of RBCs within the fibrin network in all thrombi, with no discernible higher-order structure present, such as layering. This finding is also consistent with existing knowledge as this characteristic is considered a common feature of artificial thrombi, which were used in prior imaging studies [30,31,45]. However, it should be taken into consideration that arterial thrombi in vivo are formed under complex conditions of the vascular system, involving pathophysiological interactions between the thrombus components and the endothelium. Thus, they may reveal a much more complex ultrastructure with further cellular structures, such as platelets or white blood cells [30,45,46]. Accordingly, artificially produced thrombi represent a certain discrepancy to reality in this respect, and future studies should address the extent to which the structural complexity of in vivo thrombi influences spectral CT imaging. Nevertheless, the use of artificially manufactured thrombi also offers certain advantages, as they are relatively convenient to prepare and their components can be precisely controlled. These aspects make them ideal for basic research such as conducted in prior studies, as well as in this study.

Another limitation of this study is the use of only one SCT technique, namely SDCT, and the resulting reduced generalisability of the results to other technical variants such as dual-source or kV-switching techniques [32,41]. As described initially, these differ technically from SDCT; therefore, study results may vary between devices [20,47]. Accordingly, this must naturally be taken into account when considering the results. Nevertheless, using SDCT in this context is innovative, as the spectral differentiability of thrombi in CT scans using standardised artificial thrombi has not yet been sufficiently investigated with SDCT. The majority of previous studies have examined the experimental differentiability of thrombi primarily using other techniques, and studies on SDCT are rare [31,32,41]. For example, Borggrefe et al. adopted an experimental approach to examine the uptake of iodine-containing contrast medium by different thrombi in SDCT, demonstrating that fibrin-rich thrombi absorb it more strongly [48]. However, they focused on the phenomenon of thrombus perviousness rather than on the specific tissue differentiation of thrombi in the non-contrast image using spectral maps, as was the case in the present study. This represents a completely different approach and thus differs fundamentally in terms of methodology from the existing, limited literature on this topic in SDCT [48]. Although using only one SCT technique represents a certain limitation, SDCT is a technique that has received little attention in relation to the study topic to date, despite nevertheless being clinically relevant. Accordingly, this aspect should also be positively highlighted in the present study.

The results of this study expand the knowledge on the CT-based characterisation of different thrombi and lay the groundwork for further evaluation. The next step in this context would be to validate these in vitro results in clinical studies by correlating the spectral parameters from clinical imaging with the histological characteristics of thrombectomy-removed thrombi from stroke patients and clinical parameters. While clinical imaging studies are generally available in the field of stroke research, the majority of these mainly deal with the evaluation of conventional CT density [24,35,49]. Although initial attempts have been made at conducting clinical spectral CT studies, these are rather limited and have not yet addressed the detailed investigation of ED or the dynamics of mass-attenuation curves of thrombi [44]. However, according to the results of our study, these parameters harbour particular potential that should be explored further in such clinical studies. Alongside the investigation of individual or only few imaging parameters, there are also clinical studies that examine and combine various imaging parameters in multivariate analysis models [50,51,52]. Such models are well established in other areas of imaging, but their application to thrombus characterisation is relatively new [18]. In this context, thrombus characterisation by spectral CT provides additional parameters that could be included in such analysis models, and initial approaches have been made in this regard using a limited number of parameters [44]. Yet the parameters examined in our study have not been analysed in such models, but due to their potential, as demonstrated here, they should be included and evaluated in future studies.

In conclusion, the results of this study demonstrate the innovative applicability of quantitative spectral CT parameters, such as ED or the dynamics of mass-attenuation curves, in characterising thrombi of different compositions. These parameters complement the conventional parameters that have been established to date, offering additional potential for a more detailed characterisation of thrombi using imaging techniques. The next step in this area of research is to translate these experimental results into clinical practice by incorporating spectral parameters into the clinical diagnostic algorithm for stroke imaging. Therefore, manual or automated ROI-based quantification and analysis of thrombi in the clinical imaging datasets of stroke patients could enable their pre-therapeutic characterisation. Based on this information, the most promising therapeutic approach, such as intravenous thrombolysis or a specific recanalisation technique, could then be determined. Ultimately, the goal is to establish optimised imaging that enables a more tailored, individualised therapeutic approach for each patient.

## Figures and Tables

**Table 1:** Cross-table showing the multiple comparison of conventional Hounsfield unit values between different thrombi and uncoagulated blood (5–90% and blood). *p*-values of each comparison are provided, and statistically significant results are highlighted in green. Duplicate comparisons are hidden in grey. ns: not significant.

	5%	10%	20%	30%	40%	50%	60%	70%	80%	90%	Blood
**5%**		ns	0.007	0.007	0.007	0.007	0.007	0.007	0.007	0.007	0.007
**10%**	ns		0.007	0.007	0.007	0.007	0.007	0.007	0.007	0.007	0.007
**20%**	0.007	0.007		0.01	0.007	0.007	0.007	0.007	0.007	0.007	0.007
**30%**	0.007	0.007	0.01		0.017	0.007	0.007	0.007	0.007	0.007	0.007
**40%**	0.007	0.007	0.007	0.017		ns	0.007	0.007	0.007	0.007	ns
**50%**	0.007	0.007	0.007	0.007	ns		0.019	0.007	0.007	0.007	ns
**60%**	0.007	0.007	0.007	0.007	0.007	0.019		0.008	0.007	0.007	0.007
**70%**	0.007	0.007	0.007	0.007	0.007	0.007	0.008		0.045	0.015	0.007
**80%**	0.007	0.007	0.007	0.007	0.007	0.007	0.007	0.045		ns	0.007
**90%**	0.007	0.007	0.007	0.007	0.007	0.007	0.007	0.015	ns		0.007
**Blood**	0.007	0.007	0.007	0.007	ns	ns	0.007	0.007	0.007	0.007	

**Table 2:** Cross-table showing the multiple comparison of electron density values between different thrombi and uncoagulated blood (5–90% and blood). *p*-values of each comparison are provided, and statistically significant results are highlighted in green. Duplicate comparisons are hidden in grey. ns: not significant.

	5%	10%	20%	30%	40%	50%	60%	70%	80%	90%	Blood
**5%**		ns	0.006	0.006	0.007	0.007	0.007	0.007	0.007	0.007	0.007
**10%**	ns		0.006	0.006	0.006	0.006	0.006	0.006	0.006	0.006	0.006
**20%**	0.006	0.006		0.006	0.006	0.006	0.006	0.006	0.006	0.006	0.006
**30%**	0.006	0.006	0.006		0.042	0.007	0.007	0.007	0.007	0.007	0.007
**40%**	0.007	0.006	0.006	0.042		ns	0.008	0.007	0.007	0.007	ns
**50%**	0.007	0.006	0.006	0.007	ns		ns	0.007	0.007	0.007	ns
**60%**	0.007	0.006	0.006	0.007	0.008	ns		0.027	0.007	0.007	0.021
**70%**	0.007	0.006	0.006	0.007	0.007	0.007	0.027		ns	ns	0.007
**80%**	0.007	0.006	0.006	0.007	0.007	0.007	0.007	ns		ns	0.007
**90%**	0.007	0.006	0.006	0.007	0.007	0.007	0.007	ns	ns		0.007
**Blood**	0.007	0.006	0.006	0.007	ns	ns	0.021	0.007	0.007	0.007	

**Table 3:** Cross-table showing the extent to which the relatively reduced CT density at a monoenergetic level of 200 keV compared to 40 keV allows for differentiation of the various thrombus entities and uncoagulated blood (5–90% and blood). *p*-values of each comparison are provided, and statistically significant results are highlighted in green. Duplicate comparisons are hidden in grey. ns: not significant.

	5%	10%	20%	30%	40%	50%	60%	70%	80%	90%	Blood
**5%**		ns	ns	ns	0.045	0.028	0.017	0.013	0.007	0.007	0.036
**10%**	ns		ns	ns	0.028	0.01	0.013	0.01	0.007	0.007	0.036
**20%**	ns	ns		ns	ns	ns	ns	ns	0.017	0.013	ns
**30%**	ns	ns	ns		ns	ns	ns	ns	0.007	0.007	ns
**40%**	0.045	0.028	ns	ns		ns	ns	ns	0.007	0.007	ns
**50%**	0.028	0.01	ns	ns	ns		ns	ns	ns	ns	ns
**60%**	0.017	0.013	ns	ns	ns	ns		ns	ns	ns	ns
**70%**	0.013	0.01	ns	ns	ns	ns	ns		ns	ns	ns
**80%**	0.007	0.007	0.017	0.007	0.007	ns	ns	ns		ns	ns
**90%**	0.007	0.007	0.013	0.007	0.007	ns	ns	ns	ns		0.045
**Blood**	0.036	0.036	ns	ns	ns	ns	ns	ns	ns	0.045	

## Data Availability

The datasets analysed during the current study are available on reasonable request.

## Abbreviations

The following abbreviations are used in this manuscript:

AIS	Acute ischemic stroke
RBC	Red blood cells
HU	Hounsfield units
SECT	Single-energy computed tomography
SCT	Spectral computed tomography
SDCT	Spectral detector computed tomography
ED	Electron density
Z-effective	Effective atomic number
PL	Plasma
SBI	Spectral base image
MonoE	Virtual monoenergetic
ROI	Region of interest
ROC	Receiver operating characteristic
AUC	Are under the curve
